# Screening to Identify Postoperative Pain and Cross‐Sectional Associations Between Factors Identified in This Process With Pain and Function, Three Months After Total Knee Replacement

**DOI:** 10.1002/acr.24516

**Published:** 2022-03-16

**Authors:** Vikki Wylde, Emily Sanderson, Tim J. Peters, Wendy Bertram, Nicholas Howells, Julie Bruce, Christopher Eccleston, Rachael Gooberman‐Hill

**Affiliations:** ^1^ Bristol Medical School, University of Bristol and NIHR Bristol Biomedical Research Centre, University Hospitals Bristol and Weston NHS Foundation Trust and the University of Bristol Bristol UK; ^2^ Bristol Medical School University of Bristol Bristol UK; ^3^ Bristol Medical School University of Bristol and North Bristol NHS Trust Bristol UK; ^4^ North Bristol NHS Trust Bristol UK; ^5^ University of Warwick Warwick UK; ^6^ The University of Bath Bath UK

## Abstract

**Objective:**

To describe the screening and recruitment process of a randomized trial and evaluate associations with knee pain and function 3 months after total knee replacement (TKR).

**Methods:**

In order to screen for a multicenter trial, a total of 5,036 patients were sent the Oxford Knee Score (OKS) questionnaire 10 weeks post‐TKR. Patients who reported pain in their replaced knee (score of ≤14 on the OKS pain component) completed a second OKS questionnaire 12 weeks post‐TKR. Those patients who were still experiencing pain 12 weeks post‐TKR completed a detailed questionnaire 13 weeks post‐TKR. These data were used to characterize pain in a cross‐sectional analysis. Multivariable regression was performed in order to identify factors associated with pain and function at 13 weeks post‐TKR.

**Results:**

We received OKS questionnaires from 3,058 of 5,063 TKR patients (60%), and 907 of the 3,058 (30%) reported pain in their replaced knee 10 weeks post‐TKR. By 12 weeks, 179 of 553 patients (32%) reported improved pain (score of >14 on the OKS pain component). At 13 weeks, 192 of 363 patients (53%) who completed a detailed questionnaire reported neuropathic pain, 94 of 362 (26%) reported depression symptoms, and 95 of 363 (26%) anxiety symptoms. More severe pain at 13 weeks postoperatively was associated with poorer general health, poorer physical health, more pain worry, and lower satisfaction with surgery outcome. More severe functional limitation was associated with higher levels of depression, more pain worry, lower satisfaction with surgery outcome, and higher pain acceptance.

**Conclusion:**

Screening after TKR identified individuals with pain. We identified several potential targets (physical and mental health outcomes, acceptance of pain, and quality of life) for tailored intervention to improve outcomes for patients. Future trials of multidisciplinary interventions warranted.

## INTRODUCTION

Primary total knee replacement (TKR) is a common operation, with over 100,000 operations performed in the UK's National Health Service (NHS) in 2019 (1, 2). The main indications for TKR are chronic pain and functional limitations, which are predominately related to osteoarthritis. Although the operation is successful for many, 10–34% of patients experience ongoing pain in the months and years after surgery (3). Despite its prevalence, knowledge about the onset and postoperative trajectory of chronic pain after TKR is not well understood (4). The evidence base for treatment and management is sparse (5, 6), and referrals for assessment and care are inconsistent (7, 8). There is no preoperative model as yet that can accurately predict who will have chronic pain after surgery (5, 9). People with chronic pain after TKR can feel abandoned by health care services and struggle to understand ongoing pain (10).

**Significance & Innovations**

- Our study demonstrated good uptake to early postoperative screening of pain and function after total knee replacement (TKR).
- Half of the patients with pain at 3 months after TKR reported neuropathic pain symptoms.
- One‐fourth of the patients with pain at 3 months after TKR report depression and/or anxiety.
- Multiple factors, such as quality of life, physical and mental health outcomes, and acceptance of pain, are associated with more severe pain and functional limitations after TKR, which highlights the need for multidisciplinary interventions.

The improvement trajectory following TKR is variable; however, most pain relief occurs within the first 3 months postoperatively (11). Persistent pain at 3 months could be due to slower recovery or an early indication of long‐term chronic pain. Chronic pain is difficult to treat once established (12) and the identification and characterization of pain early in the recovery trajectory could facilitate the delivery of targeted interventions to support recovery and improve long‐term pain outcomes. Hence, there is potential for early identification of these patients to explore whether intervention is warranted.

Previous studies have described pain after TKR (3, 13, 14, 15, 16, 17), but these studies have methodologic shortcomings that have contributed to the poor quantification and characterization of pain after TKR. These shortcomings include the use of surgeon‐administered tools to assess pain, limited assessment of the multidimensional nature of pain, variable definitions of pain resulting in different prevalence estimates, and single‐center studies, which all limit generalizability (3, 16, 18). A robust method of identifying patients with pain after TKR using the Oxford Knee Score (OKS) pain component has been developed (19). Using data from a national population‐based cohort across England, patients with a postoperative score of ≤14 on the OKS pain component were identified as having pain likely to negatively impact health‐related quality of life (19). Applying this method for identification of patients with pain in the first 3 months postoperatively allows the early investigation of pain characteristics. The aim here is to describe our screening procedures to identify people with postoperative pain and to identify associations with pain and function among patients with pain in the first 3 months after primary TKR.

## PATIENTS AND METHODS

### Design

The data analyzed in the present study are from the Support and Treatment After Joint Replacement (STAR) trial, a multicenter randomized trial evaluating the effectiveness of a care pathway for patients with chronic pain at 3 months after TKR (20). Screening data included in the analyses of the present study were collected before randomization and were analyzed as observational data. Study methods relevant to these analyses were described and reported following the Strengthening and Reporting of Observational Studies in Epidemiology guidance (see Supplementary Table 1, available on the *Arthritis Care & Research* website at http://onlinelibrary.wiley.com/doi/10.1002/acr.24516).

### Patient and public involvement

This research was conducted in collaboration with the Patient Experience Partnership in Research STAR group, which is a specialized group comprised of 5 patients who have experienced chronic pain after TKR. Through regular group meetings, patient representatives contributed to project design and management.

### Participants

Between September 2016 and May 2019, eligible patients were recruited into the STAR trial from 8 NHS orthopedic centers in Bristol, Cardiff, Exeter, Mansfield, Oswestry, Wrightington, Leicester, and Birmingham. Inclusion criteria included adults who received a primary TKR for osteoarthritis and reported pain in their replaced knee 12 weeks postoperatively. Exclusion criteria included lack of capacity to provide informed consent, previous study participation for the contralateral knee, or participation in another project that interfered with the STAR trial. The STAR trial complied with the Declaration of Helsinki and was approved by the Southwest–Central Bristol Research Ethics Committee (16/SW/0154) and the Health Research Authority. All participants provided written informed consent in 2 stages: 1) for the screening study only, comprising OKS measurements at 10 and 12 weeks after TKR and 2) for the main STAR trial, comprising a detailed baseline questionnaire at 13 weeks after TKR. Identification of patients with pain after TKR began 10 weeks postoperatively to ensure timely identification of those with pain that persisted 3 months postoperatively. We reported our findings of screening procedures and the cross‐sectional analysis of associations with pain and function 13 weeks after TKR surgery.

### Initial postal screening to identify patients with pain 10 weeks after TKR

Patients who received a primary TKR due to osteoarthritis 8 weeks previously were sent a study information leaflet, consent form, and short initial screening questionnaire, including the OKS (21) and sociodemographic questions. Nonresponders received a single reminder. The OKS is a joint‐specific measure of pain and function consisting of 12 items with 5 ordinal response options for each item (21). There is evidence of validity and reliability, with the OKS being reported as the best performing site‐specific patient‐reported outcome measure in a psychometric review of 32 measures used in hip and knee replacement surgery (22). It has an overall score ranging from 0–48 (worst to best). Two subscales can be calculated, including a 5‐item OKS function component (raw score of 0–20) and a 7‐item OKS pain component (raw score of 0–28). Patients with a score of 0–14 on the raw OKS pain component were considered to have pain that was likely to negatively impact health‐related quality of life (19). It is recommended that the component scores are standardized to a 0–100 scale (worst to best) for analysis (23).

### Second telephone screening to confirm ongoing pain 12 weeks after TKR

All responding patients who reported an OKS pain score of ≤14 at 10 weeks were contacted by telephone at 12 weeks and invited to complete a second screening questionnaire that repeated the OKS questionnaire, in order to confirm their pain status. Those still reporting clinically meaningful pain (defined as an OKS pain score of ≤14) at 12 weeks were eligible for invitation to enter the trial.

### Detailed study questionnaire at 13 weeks after TKR for patients with pain

Participants who gave their consent to the trial completed a third OKS questionnaire as part of a more detailed study questionnaire prior to randomization. If questionnaires were not returned within 1 week, the participant was offered support on the telephone with a researcher.

The outcomes assessed in the questionnaire administered 13 weeks postoperatively reflected the 8 domains of the core outcome set for chronic pain after TKR (24). Pain severity and pain interference were assessed using the Brief Pain Inventory (BPI) (subscale scores range 0–10 [best to worst]) (25). Knee pain and function were measured using the OKS questionnaire.

Pain with neuropathic features was assessed using 2 questionnaires. First, we used the PainDETECT (26), which can be analyzed as a continuous score (range –1 to 38, with a higher score indicating greater likelihood of neuropathic pain) or categorized into nociceptive pain (range –1 to 12), possible neuropathic pain component (range 13–18), or probable neuropathic pain component (range 19, 20, 21, 22, 23, 24, 25, 26, 27, 28, 29, 30, 31, 32, 33, 34, 35, 36, 37, 38). Second, the Dolour Neuropathic scale (DN4) (27), with scores ranging from 0–7 (best to worst) and a score of ≥3 indicating neuropathic pain characteristics, was used. Single questions evaluated the frequency of pain in the past 24 hours and 4 weeks and how this pain compared to preoperative pain.

General health was measured using the Short Form 12 (SF‐12) health survey (28), comprising a physical component score and a mental component score (range 0–100 [worst to best]). Health‐related quality of life was assessed by the 5‐level version of the EuroQol 5‐domain instrument (EQ‐5D‐5L [29]) (range –0.594 to 1, where 1 indicates “perfect health” and 0 indicates “dead”). Capability was assessed by the Icepop Capability Measure for Adults (ICECAP‐A [30]) (range –0.001 to 1 [worst to best]).

Depression and anxiety were assessed using the Hospital Anxiety and Depression Scale (HADS) (31), with subscale scores (HADS anxiety and HADS depression) ranging from 0 to 21 (best to worst) and categorized into unlikely symptoms of depression/anxiety (range 0–7), possible depression/anxiety (range 8, 9, 10), and probable depression/anxiety (range 11, 12, 13, 14, 15, 16, 17, 18, 19, 20, 21). Worry about pain was assessed with the Pain Catastrophizing Scale (PCS) (range 0–52 [best to worst]) (32), which consists of 3 subscales labeled rumination (scored 0–16), magnification (scored 0–12), and helplessness (scored 0–24). The Possible Solutions to Pain Questionnaire (33) was also completed, and the 4 subscales were analyzed, including solving pain (scored 0–24 [worst to best]), meaningfulness of life despite pain (scored 0–30), acceptance of the insolubility of pain (scored 0–18), and belief in solutions (scored 0–12).

Patient satisfaction with the outcome of surgery was measured by the Self‐Administered Patient Satisfaction Scale (34), a 4‐item arthroplasty‐specific score (range 25–100 [worst to best]). Painful body regions were indicated on a body diagram, and widespread pain was defined as pain in at least 2 sections of each 2 contralateral arms or legs and in the axial skeleton (35). Sociodemographic questions included age, sex, marital and living status, ethnicity, and education level.

### Statistical analysis

#### Screening questionnaire

In addition to response rates, distributions of screening OKS scores were assessed at each phase using histograms and summary statistics, such as the mean ± SD. Regression analyses were performed on the OKS pain and function subscores as the outcome variables to explore the associations with age and sex. Results are shown as regression coefficients, 95% confidence intervals, and *P* values. The relationship between the OKS subscales were assessed using scatter plots, replicated stratified by age group (age ranges of <60, 60–70, 71–80, and >80 years) and sex.

#### Study questionnaire analyses

Summary statistics for sociodemographic data and patient‐reported outcomes were presented as mean ± SD, median (interquartile range), and number (%). Distributions of the OKS scales were presented as histograms, in order to assess normality. Correlation coefficients between pain outcomes were evaluated. Linear regression was used to evaluate the factors independently associated with the OKS pain and function scores. Staged regression was then used to select variables systematically for the linear regression model (36). Associations were explored between OKS pain and groups of factors, including sociodemographic variables, general health, and mental health measures. Each group of variables was first explored separately in multivariable regression models, with (iterative) exclusion of variables without strong associations with OKS pain when adjusted for other variables in the model. The process was then extended to consider all groups together, resulting in a final regression model containing only variables that were strongly associated with OKS pain, adjusted for other variables. This process was repeated for OKS function exploring associations with sociodemographic variables and mental health outcomes. In all analyses, the standardized OKS pain and function scores (range 0–100) were used (23).

Data completeness is reported in the Tables and Figures. For the OKS component scores, the mean of other items on the subscale was used to impute a missing item, if only 1 item was missing. If more than 1 item was missing, a score was not calculated (37). The approach to missing data for other validated questionnaires followed guidance recommended by the questionnaire developers; further details are in the STAR trial statistical analysis plan (38).

#### Sample size

The sample size for the STAR trial was based on detecting a minimal clinically important difference between trial arms in the BPI subscales 12 months after randomization (20). We did not undertake a separate power calculation for the analyses presented herein, as our intention was to investigate characteristics of the study population collected prior to randomization; rather, the levels of achieved precision are indicated through the relevant confidence intervals.

### Data availability

The data sets generated during the current study will be available in the University of Bristol Research Data Repository (https://data.bris.ac.uk/data/). Data will be available following publication of the trial results. Access to the data will be restricted to ensure that data is only made available to bona fide researchers for ethically approved research projects, on the understanding that confidentiality will be maintained and after a data access agreement has been signed by an institutional signatory.

## RESULTS

### Recruitment, screening, and participant flow

**Figure 1:**
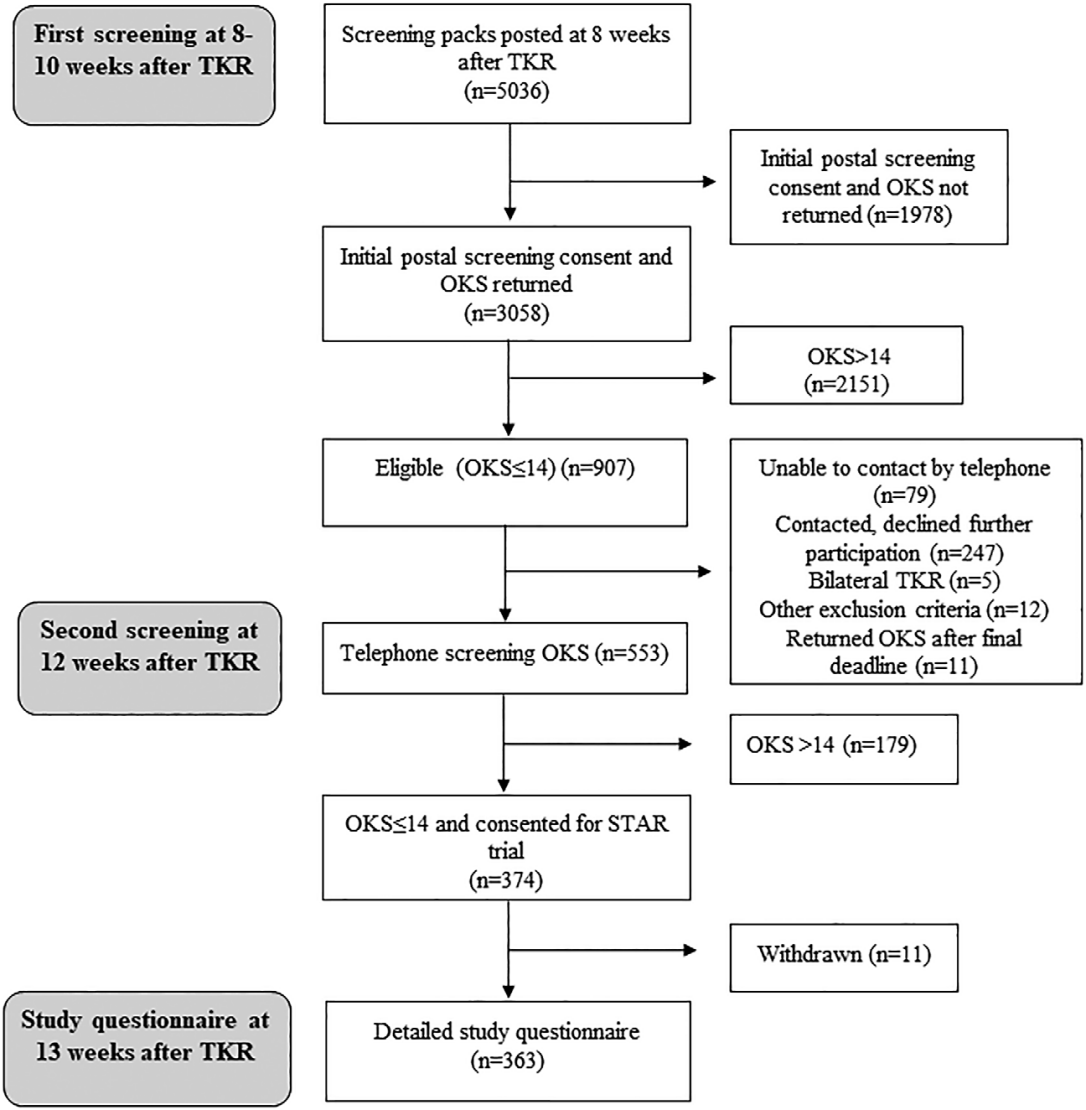
Participant flow chart. TKR = total knee replacement; OKS = Oxford Knee Score; STAR = Support and Treatment After Joint Replacement.

An overview of participant flow through the study is provided in Figure 1. Screening questionnaires to identify patients with pain after TKR were sent to 5,036 patients who had a TKR at 1 of 8 orthopedic centers. Completed screening questionnaires were returned postoperatively by 3,058 patients (61%) at a mean ± SD of 10 ± 2 weeks. Of these patients, 907 (30%) reported pain in their replaced knee at 10 weeks, of whom 553 (61%) completed a second telephone OKS to confirm pain status at a mean ± SD of 12 ± 2 weeks. The mean ± SD age of the 553 patients who completed a telephone questionnaire was 67.7 ± 8.6 years, with 56% of the patients being female. Those who did not complete a telephone questionnaire at 12 weeks (n = 354) were slightly older, with a mean ± SD age of 69.4 ± 10.4 years, and 62% were female. Patients who completed the 12‐week telephone‐administered OKS questionnaire had a slightly higher mean ± SD OKS score (18.2 ± 5.4) than those who did not complete a telephone OKS questionnaire (17.2 ± 5.7), indicating less pain and better function in responders compared with nonresponders at 12 weeks. A total of 363 of 553 patients (66%) completed a detailed questionnaire at a mean ± SD 13 ± 2 weeks.

Sociodemographic characteristics of responders and nonresponders to the screening questionnaire at 10 weeks are provided in Table 1. The mean age was comparable (70 years), although female patients were slightly less likely to respond than male patients (55% versus 62%). The OKS overall and component scores are in Table 1 and Supplementary Figures 1 and 2 (available on the *Arthritis Care & Research* website at http://onlinelibrary.wiley.com/doi/10.1002/acr.24516). Overall, 907 of 3,058 patients (30%) reported clinically meaningful pain in their replaced knee at 10 weeks (OKS pain component score ≤14).

**Table 1:** Characteristics of responders and nonresponders to screening questionnaire at 10 and 12 weeks post‐TKR*

	Responders	Nonresponders
Screening questionnaire 10 weeks post‐TKR		
Total, no. (%)	3,058 (61)	1,977 (39)
Age, years	69.7 ± 8.8	69.9 ± 9.8
Female sex, %	54.5	62.2
Total OKS (0–48; worst to best)	29.3 ± 9.6	–
OKS pain component (0–100; worst to best)	62.3 ± 21.2	–
OKS function component (0–100; worst to best)	59.4 ± 20.8	–
Telephone‐administered screening questionnaire at 12 weeks post‐TKR†		
Total, no. (%)	553 (61)	354 (39)
Age, years	67.7 ± 8.6	69.4 ± 10.4
Female sex, %	56.2	62.0
Total OKS 10 weeks post‐TKR	18.2 ± 5.4	17.2 ± 5.7
OKS pain component 10 weeks post‐TKR	36.6 ± 11.3	35.6 ± 12.2
OKS function component 10 weeks post‐TKR	40.0 ± 14.4	36.2 ± 15.0

* Values are the mean ± SD unless indicated otherwise. OKS = Oxford Knee Score; TKR = total knee replacement.

† Eligible at 10 weeks (n = 907).

Scatter plots of the OKS component scores demonstrated a linear relationship between pain and function, with similar patterns when stratified by sex and age (see Supplementary Figures 3–5, available at http://onlinelibrary.wiley.com/doi/10.1002/acr.24516). Younger age and female sex were associated with worse knee pain severity and functional limitations at 10 weeks (Table 2).

**Table 2:** Univariable associations between age, sex, pain, and function 10 weeks after TKR*

		OKS pain component		OKS function component	
		Coefficient		Coefficient	
	No.	(95% CI)	*P*	(95% CI)	*P*
Age	2,915	0.29 (0.20, 0.37)	<0.001	0.09 (0.009, 0.18)	0.030
Sex (ref. = male)	3,042	–3.09 (–4.60, –1.58)	<0.001	–7.18 (–8.64, –5.71)	<0.001

* 95% CI = 95% confidence interval; OKS = Oxford Knee Score; ref. = reference; TKR = total knee replacement.

Of the 533 of 907 patients to complete the OKS by telephone, 179 (32%) reported an improvement in pain (OKS score of >14), but 374 (68%) remained in pain. A summary of the statistics of age, sex, and week‐10 OKS scores for those who did and did not respond at week 12 are presented in Table 1. Responders were slightly younger than nonresponders at 12 weeks, with a lower proportion of female responders. Responders at 12 weeks had slightly higher week‐10 OKS scores compared with those who did not respond at 12 weeks.

### Characterization of people reporting pain 13 weeks post‐TKR

Sociodemographic characteristics and patient‐reported outcomes for the 363 of 374 (97%) participants who completed a detailed questionnaire at 13 weeks are shown in Table 3. The mean ± SD age of these participants was 67 ± 9 years, and 60% were female. Neuropathic pain characteristics were common, with half of the participants (53%) having a PainDETECT score that indicated likely neuropathic pain (score >19) and three‐fourths (74%) of patients having neuropathic pain characteristics according to the DN4 (score of ≥3). A total of 47% of patients likely had both neuropathic pain according to PainDETECT and neuropathic pain according to the DN4. Poor mental health was also common, with patients having HADS scores indicative of either probable depression (26%) or anxiety (26%); of these patients, 60 of 362 (17%) reported symptoms of both depression and anxiety. Over the previous 4 weeks, 96% of patients had experienced pain frequently, defined as pain being present “often,” “most of the time,” or “all the time.” Almost half of patients (44%) reported their pain as “a bit worse” or “much worse” than their preoperative pain. Despite still being in pain at 12 weeks, most patients (74%) were satisfied with their overall outcome from TKR, and 55% were satisfied with their pain relief, although satisfaction rates with ability to do activities of daily living and leisure activities were lower (39% and 38%, respectively).

**Table 3:** Characteristics of participants with pain 3 months after TKR (n = 363)*

Characteristic	
Age, years	
Mean ± SD	67.2 ± 8.7
Median (IQR)	67 (61–73)
Range	40–88
Sex	
Female	217 (60)
Male	146 (40)
Marital status (n = 356)	
Single	25 (7)
Married/partner	251 (71)
Divorced/separated	35 (10)
Widowed	45 (13)
Living arrangement (n = 356)	
Live alone	78 (22)
With spouse/partner	253 (71)
With someone else	22 (6)
Other	3 (1)
Ethnicity (n = 356)	
White	335 (94)
Asian	13 (4)
Black	5 (1)
Mixed	1 (<1)
Other	2 (<1)
Education level (n = 318)	
School left <16 years	22 (7)
School left 16 years	194 (61)
College	63 (20)
University	15 (5)
Other postgraduate	24 (8)
BPI scores, mean ± SD	
Severity	5.2 ± 1.7
Interference	6.28 ± 1.92
OKS scores, mean ± SD	
Total	18.23 ± 5.83
Pain	36.75 ± 12.70
Function	39.70 ± 14.28
Pain Catastrophizing Scale, median (IQR) (n = 360)	
Total	18 (9.25–30.5)
Rumination	8 (4, 5, 6, 7, 8, 9, 10, 11, 12)
Magnification	2 (1, 2, 3, 4, 5)
Helplessness	8 (4, 5, 6, 7, 8, 9, 10, 11, 12, 13, 14)
Pain solution (PaSol), median (IQR)	
Solving pain (n = 362)	18 (14, 15, 16, 17, 18, 19, 20, 21, 22)
Meaningful life (n = 362)	22 (18, 19, 20, 21, 22, 23, 24, 25, 26)
Acceptance of the insolubility of pain (n = 358)	8 (5, 6, 7, 8, 9, 10, 11)
Belief in solution (n = 359)	9 (6, 7, 8, 9, 10, 11, 12)
Patient Satisfaction, mean ± SD (n = 360)	62.88 ± 18.99
ICECAP‐A, median (IQR) (n = 362)	0.78 (0.55–0.89)
SF‐12, mean ± SD	
Physical score	33.44 ± 6.51
Mental score	42.19 ± 11.12
EQ‐5D‐5L, median (IQR) (n = 358)	0.53 (0.30–0.62)
DN4 score, mean ± SD (n = 359)	3.79 ± 1.71
Neuropathic pain characteristics according to DN4?	
Yes	267 (74)
No	92 (26)
PainDETECT score, mean ± SD	18.19 ± 6.77
Neuropathic pain characteristics according to PainDETECT?	
Unlikely	76 (21)
Ambiguous	96 (26)
Likely	191 (53)
HADS: Anxiety	
Normal	197 (54)
Borderline anxiety	71 (20)
Clinical anxiety	95 (26)
HADS: Depression (n = 362)	
Normal	177 (49)
Borderline depression	91 (25)
Clinical depression	94 (26)
Pain frequency in past 24 hours (n = 361)	
Rarely	1 (<1)
Sometimes	40 (11)
Often	98 (27)
Most of the time	164 (45)
All of the time	58 (16)
Pain frequency in past 4 weeks (n = 362)	
Rarely	0 (0)
Sometimes	14 (4)
Often	102 (28)
Most of the time	156 (43)
All of the time	90 (25)
Satisfaction with overall results of TKR (n = 359)	
Very dissatisfied	21 (6)
Somewhat dissatisfied	72 (20)
Somewhat satisfied	154 (43)
Very satisfied	112 (31)
Satisfaction with improving pain (n = 359)	
Very dissatisfied	47 (13)
Somewhat dissatisfied	117 (33)
Somewhat satisfied	139 (39)
Very satisfied	56 (16)
Satisfaction with improving ability to do housework or gardening (n = 358)	
Very dissatisfied	65 (18)
Somewhat dissatisfied	152 (42)
Somewhat satisfied	111 (31)
Very satisfied	30 (8)
Satisfaction with improving ability to do leisure activities (n = 359)	
Very dissatisfied	86 (24)
Somewhat dissatisfied	140 (39)
Somewhat satisfied	106 (30)
Very satisfied	27 (8)
Comparison of pain to preoperative pain (n = 362)	
Much better	79 (22)
A bit better	70 (19)
The same	54 (15)
A bit worse	77 (21)
Much worse	82 (23)
Presence of chronic widespread pain (Manchester definition)	
Yes	16 (4)
No	347 (96)

* Values are the number (%) unless indicated otherwise. BPI = Brief Pain Inventory; DN4 = Dolour Neuropathic scale; EQ‐5D‐5L = 5‐level version of the EuroQol 5‐domain instrument; HADS = Hospital Anxiety and Depression Scale; ICECAP‐A = Icepop Capability Measure for Adults; IQR = interquartile range; OKS = Oxford Knee Score; PaSOL = Pain Solutions Questionnaire; SF‐12 = Short Form 12 health survey; TKR = total knee replacement.

#### Regression analysis

Results of the linear regression model with the OKS pain component as the outcome are shown in Table 4. In this cross‐sectional analysis, having more severe knee pain at 13 weeks was associated with lower general health measured by the EQ‐5D‐5L utility score, lower physical health measured by the SF‐12 health survey, higher pain worry (PCS), and lower satisfaction with the outcome of surgery.

**Table 4:** Final model from the linear regression for associations with pain 3 months after TKR (n = 352)*

Variable	Coefficient (95% CI)	*P*
EQ‐5D‐5L	19.9 (14.1, 25.8)	<0.001
SF‐12 (physical)	0.25 (0.09, 0.42)	0.003
Pain Catastrophizing Scale	–0.27 (–0.36, –0.17)	<0.001
Patient Satisfaction Scale	0.11 (0.05, 0.16)	<0.001

* 95% CI = 95% confidence interval; EQ‐5D‐5L = 5‐level version of the EuroQol 5‐domain instrument; SF‐12 = Short Form 12 health survey.

From the linear regression model with the OKS function component as the outcome (Table 5), in patients with pain at 13 weeks postoperatively, more severe functional limitation was associated with higher levels of depression, higher pain catastrophizing, lower satisfaction with the outcome of surgery, and higher levels of acceptance of the insolubility of pain.

**Table 5:** Final model from the linear regression for associations with function 3 months after TKR (n = 353)*

Variable	Coefficient (95% CI)	*P*
HADS depression	–1.18 (–1.55, –0.80)	<0.001
PaSOL (acceptance of pain)	–0.52 (–0.78, –0.25)	<0.001
Pain Catastrophizing Scale	–0.24 (–0.36, –0.12)	<0.001
Patient Satisfaction Scale	0.09 (0.01, 0.16)	0.019

* 95% CI = 95% confidence interval; HADS = Hospital Anxiety and Depression Scale; PaSOL = Pain Solutions Questionnaire; TKR = total knee replacement.

## DISCUSSION

The present study examined characteristics of people reporting pain 10 to 13 weeks after TKR. We used the validated OKS questionnaire pain component threshold to identify patients with pain in the first 3 months after TKR. Using this standardized pain definition, 30% of patients reported pain in their replaced knee 10 weeks after surgery. Of the 553 patients who completed a second OKS questionnaire by telephone (12 weeks after TKR), 30% reported an improvement in their OKS pain score from the 10‐week measurement. However, for the majority (70%), the pain was still present at 3 months. Applying the OKS pain threshold allowed an in‐depth evaluation of the characteristics of patients with pain 3 months after TKR. We found that more than half of the patients reported pain with neuropathic characteristics, one‐fourth of the patients reported probable depression or anxiety, and 17% reporting both depression and anxiety. Despite still having problems with pain, three‐fourths of these patients were satisfied with their TKR outcome. Patients with more severe knee pain at 3 months were likely to have poorer general health, poorer physical health, higher pain worry (measured as pain catastrophizing), and lower satisfaction with the outcome of surgery. Patients with greater functional limitations were more likely to have higher levels of depression, higher pain worry, lower satisfaction with the outcome of surgery, and higher levels of acceptance of the pain's insolubility.

Previously, the lack of a robust approach to screening has been a barrier to the implementation of new services to improve care for patients with pain after TKR (7). Our study demonstrated that early screening using the OKS questionnaire definition of chronic pain as a standardized approach to identify patients with pain is achievable. In our large multicenter trial, one‐third of patients met our definition of pain at 10 weeks; this is not unexpected, as TKR has a long recovery period and individual patients’ recovery trajectories vary (11). One‐third of patients with pain at 10 weeks had improved by 12 weeks, demonstrating that patients can experience rapid recovery during this early postoperative period. However, 70% of responding patients with pain at 10 weeks still had pain at 12 weeks, and for some, this pain is likely to persist for the long term. Early screening to identify patients with pain at 3 months could facilitate targeted care delivery (e.g., through transitional pain clinics) to prevent the transition of acute pain to chronic pain (39).

The prevalence of neuropathic pain after TKR and other types of surgery differs in the literature, likely due to variation in definition and measurement (40). This warrants further research and suggests a potential role for routine screening and treatment of neuropathic pain after TKR. A systematic review by Finnerup et al has identified inadequate response to pharmacotherapy for neuropathic pain, which relates to modest efficacy, high placebo rates, and poor phenotyping (41). Further work could examine the development of targeted interventions including nonpharmacologic treatments. For example, the National Institute for Health and Care Excellence currently recommends trials comparing the effectiveness of combination therapy versus monotherapy for neuropathic pain (42). Another potential target for intervention is depression and anxiety, reported by one‐fourth of participants in our study. Given the known association between mental health and chronic pain (43), concurrent treatment of both conditions may improve outcomes for patients.

An interesting finding in the present study was that, despite ongoing pain, satisfaction with treatment was high. This may have been in part influenced by the relatively early time point of assessment postsurgery and an acceptance that initial postoperative pain is part of the recovery trajectory. Satisfaction is a complex construct that can be influenced by a wide array of interrelated factors (44). The degree of dissatisfaction experienced by patients with chronic pain after TKR has been associated with various factors, including instability in the coronal plane, stiffness, and negative social support (17). Further research would help to further understand the factors that influence patients’ satisfaction with their outcome.

Our analysis also identified factors that were associated with more severe pain and functional limitations at 3 months. These associations are consistent with previous studies of pain conditions (44, 45, 46) and present potential areas for intervention to improve patient outcomes. Any such intervention should be multidisciplinary to address the varied nature of factors associated with pain. The association of more severe functional limitations with higher levels of pain acceptance of the insolubility of pain that is beyond the general measures of mental health is highly unusual and deserves further attention. This association might be artifactual (floor effect), as 27% of the sample recorded “not applicable” to the item “I can accept that there is no solution for my pain.” Many patients found the idea of accepting the lack of a solution as simply not relevant to their early postoperative phase. The association could be explained by some patients entertaining the idea of accepting the insolubility of pain because of severity of symptoms. Speculatively, it could also demonstrate a fatalistic coping strategy in which one expects pain after surgery. This coping style could be negative, acting as a barrier to engaging with treatment‐seeking for pain, or could be positive, acting as a means to disengage from unachievable goals (47).

There are several factors limiting the interpretation of the results from this study. First, the response rate of 61% to the initial postal screening questionnaire at 10 weeks, although comparable to other surveys of orthopedic populations (17, 48), may have introduced a responder bias (49). Of note, women were slightly less likely to respond to the screening questionnaire, and female sex was associated with more severe pain at 10 weeks; this may underestimate pain prevalence. Second, our screening of patients with pain after TKR began 10 weeks postoperatively, sooner than the internationally accepted 3‐month definition of chronic postsurgical pain (50). This approach was necessary to ensure the timely identification of patients with postoperative pain at 3 months. Treatment of pain becomes more difficult once pain is established and becomes chronic. Our study demonstrates that identification of patients with pain early in the recovery trajectory is feasible to undertake (12). Third, the data were cross‐sectional so the direction of effects could not be determined. Fourth, our study sample was limited to those with pain 3 months after TKR, which limits the generalizability of our results. When interpreting the baseline factors associated with pain and function, we cannot know if these associations are unique to those with pain 3 months after TKR. Such interpretation is further limited by the lack of preoperative data on our patient cohort, which was not feasible to obtain but would allow further examination of those at higher risk of post‐TKR pain. Finally, the measurement tools limit interpretation; although the PainDETECT and DN4 are widely used self‐report screening tools for pain with neuropathic characteristics, a detailed clinical examination is recommended to confirm diagnosis (51).

In conclusion, large‐scale early screening after TKR identified ongoing pain in a relatively high proportion of people who may benefit from tailored intervention to prevent chronicity. Our study found a high prevalence of pain with neuropathic characteristics and identified several potential intervention targets to improve outcomes for patients with pain at 3 months post‐TKR. Research is needed to build on our findings and evaluate multidisciplinary and targeted interventions to improve outcomes for people with pain after TKR.

## AUTHOR CONTRIBUTIONS

All authors were involved in drafting the article or revising it critically for important intellectual content, and all authors approved the final version to be submitted for publication. Dr. Wylde had full access to all of the data in the study and takes responsibility for the integrity of the data and the accuracy of the data analysis.

### Study conception and design

Wylde, Peters, Howells, Bruce, Eccleston, Gooberman‐Hill.

### Acquisition of data

Bertram.

### Analysis and interpretation of data

Sanderson, Peters.

## Supporting information


**Appendix S1**: Supplementary Information
