# Supplementary material for: Screening to Identify Postoperative Pain and Cross‐Sectional Associations Between Factors Identified in This Process With Pain and Function, Three Months After Total Knee Replacement
Source: Arthritis Care Res (Hoboken). 2022 Mar 16;74(5):790–8. doi: 10.1002/acr.24516 (PMC9311148; doi:10.1002/acr.24516)
Supplement: Supplementary file 1 — Appendix S1: Supplementary Information [file ACR-74-790-s001.docx]

**Supplementary Table 1: STROBE checklist for cohort studies**

|  | Item No | Recommendation | Section in paper |
| --- | --- | --- | --- |
| **Title and abstract** | 1 | (*a*) Indicate the study’s design with a commonly used term in the title or the abstract | Title |
|  |  | (*b*) Provide in the abstract an informative and balanced summary of what was done and what was found | Abstract |
| Introduction | | |  |
| Background/rationale | 2 | Explain the scientific background and rationale for the investigation being reported | Introduction |
| Objectives | 3 | State specific objectives, including any prespecified hypotheses | Introduction |
| Methods | | |  |
| Study design | 4 | Present key elements of study design early in the paper | Methods: design section |
| Setting | 5 | Describe the setting, locations, and relevant dates, including periods of recruitment, exposure, follow-up, and data collection | Methods: participants, screening questionnaire and detailed questionnaire sections |
| Participants | 6 | (*a*) Give the eligibility criteria, and the sources and methods of selection of participants. Describe methods of follow-up | Methods: participants section |
|  |  | (*b*) For matched studies, give matching criteria and number of exposed and unexposed | N/A |
| Variables | 7 | Clearly define all outcomes, exposures, predictors, potential confounders, and effect modifiers. Give diagnostic criteria, if applicable | Methods: screening questionnaire and detailed questionnaire sections |
| Data sources/ measurement | 8 | For each variable of interest, give sources of data and details of methods of assessment (measurement). Describe comparability of assessment methods if there is more than one group | Methods: screening questionnaire and detailed questionnaire sections |
| Bias | 9 | Describe any efforts to address potential sources of bias | Methods: screening questionnaire and detailed questionnaire sections |
| Study size | 10 | Explain how the study size was arrived at | Methods; sample size section |
| Quantitative variables | 11 | Explain how quantitative variables were handled in the analyses. If applicable, describe which groupings were chosen and why | Methods: screening questionnaire, detailed questionnaire and statistical analysis sections |
| Statistical methods | 12 | (*a*) Describe all statistical methods, including those used to control for confounding | Methods: statistical analysis section |
|  |  | (*b*) Describe any methods used to examine subgroups and interactions | Methods: statistical analysis section |
|  |  | (*c*) Explain how missing data were addressed | Methods: statistical analysis section |
|  |  | (*d*) If applicable, explain how loss to follow-up was addressed | N/A |
|  |  | (*e*) Describe any sensitivity analyses | N/A |
| Results | | |  |
| Participants | 13 | (a) Report numbers of individuals at each stage of study—eg numbers potentially eligible, examined for eligibility, confirmed eligible, included in the study, completing follow-up, and analysed | Results: Recruitment and participant flow, Figure 1 |
|  |  | (b) Give reasons for non-participation at each stage | Figure 1 |
|  |  | (c) Consider use of a flow diagram | Figure 1 |
| Descriptive data | 14 | (a) Give characteristics of study participants (eg demographic, clinical, social) and information on exposures and potential confounders | Results: Identification of patients with pain, characterisation of pain at 3 months  Table 1 |
|  |  | (b) Indicate number of participants with missing data for each variable of interest | Table 3 |
|  |  | (c) Summarise follow-up time (eg, average and total amount) | Results: Recruitment and participant flow |
| Outcome data | 15 | Report numbers of outcome events or summary measures over time | Table 3 |
| Main results | 16 | (*a*) Give unadjusted estimates and, if applicable, confounder-adjusted estimates and their precision (eg, 95% confidence interval). Make clear which confounders were adjusted for and why they were included | Tables 2-5 |
|  |  | (*b*) Report category boundaries when continuous variables were categorized | Table 3 |
|  |  | (*c*) If relevant, consider translating estimates of relative risk into absolute risk for a meaningful time period | N/A |
| Other analyses | 17 | Report other analyses done—eg analyses of subgroups and interactions, and sensitivity analyses | Results: Identification of patients with pain |
| Discussion | | |  |
| Key results | 18 | Summarise key results with reference to study objectives | Discussion: First paragraph |
| Limitations | 19 | Discuss limitations of the study, taking into account sources of potential bias or imprecision. Discuss both direction and magnitude of any potential bias | Discussion: Second paragraph |
| Interpretation | 20 | Give a cautious overall interpretation of results considering objectives, limitations, multiplicity of analyses, results from similar studies, and other relevant evidence | Discussion: Third and fourth paragraph |
| Generalisability | 21 | Discuss the generalisability (external validity) of the study results | Discussion: Second paragraph |
| Other information | | |  |
| Funding | 22 | Give the source of funding and the role of the funders for the present study and, if applicable, for the original study on which the present article is based | Funding acknowledgement |

**
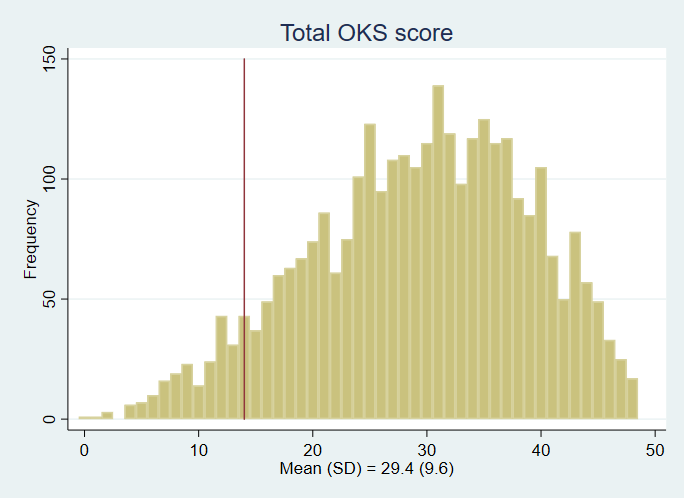
**

**Supplementary Figure 1:** **Distribution and descriptive statistics of the OKS total scores at 10 weeks after TKR (n=3054) including vertical line at cut-off for having sufficient pain to be included (OKS = 14)**

**
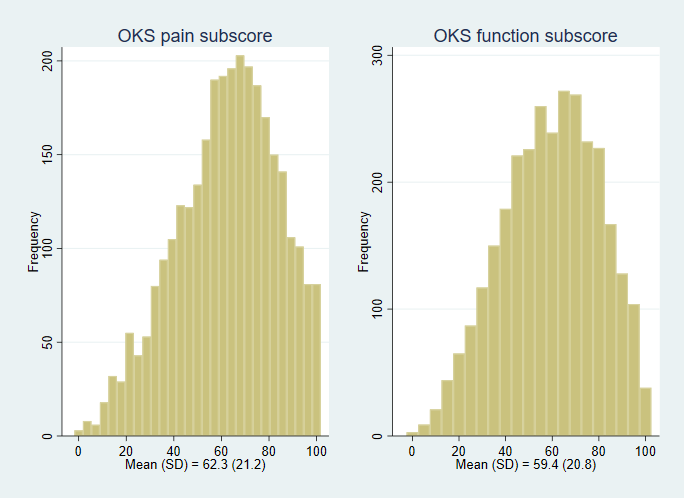
**

**Supplementary Figure 2:** **Distribution and descriptive statistics of the OKS pain and function component scores at 10 weeks after TKR (n=3054)**


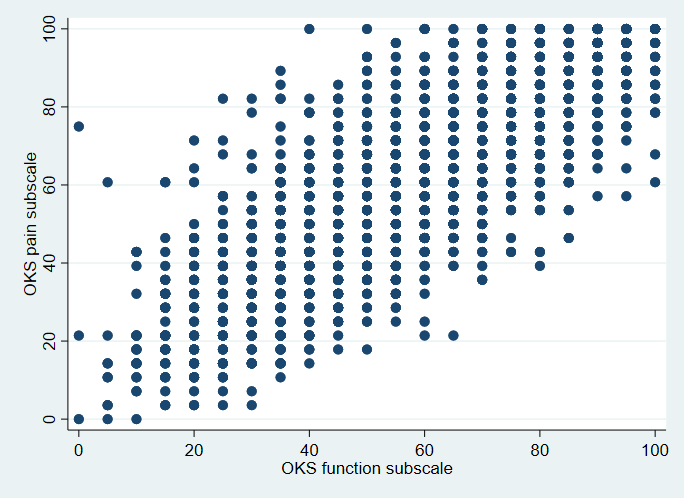


**Supplementary Figure 3: Scatterplot of the OKS pain and function component scores**


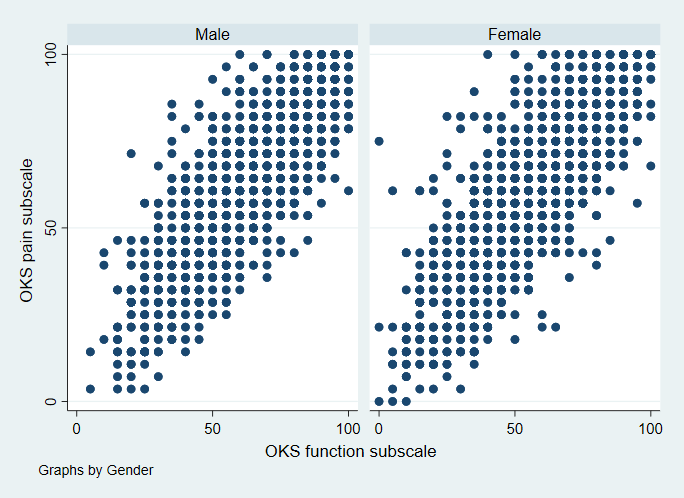


**Supplementary Figure 4: Scatterplot of the OKS pain and function components by gender**


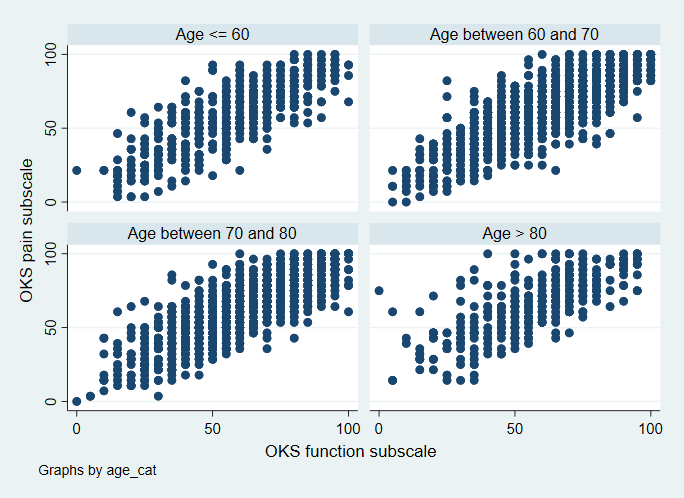


**Supplementary Figure 5: Scatterplot of the OKS pain and function components by age**

**Supplementary Table 2: correlation coefficients between pain outcomes**

|  | BPI severity | BPI interference | OKS score | OKS pain subscore | OKS function subscore | DN-4 | PainDETECT |
| --- | --- | --- | --- | --- | --- | --- | --- |
| BPI severity | 1 |  |  |  |  |  |  |
| BPI interference | 0.586 | 1 |  |  |  |  |  |
| OKS score | -0.5759 | -0.7147 | 1 |  |  |  |  |
| OKS pain subscore | -0.5817 | -0.6769 | 0.926 | 1 |  |  |  |
| OKS function subscore | -0.4486 | -0.6124 | 0.8831 | 0.6407 | 1 |  |  |
| DN-4 | 0.3166 | 0.2635 | -0.2644 | -0.2468 | -0.2309 | 1 |  |
| PainDETECT | 0.5042 | 0.3957 | -0.4517 | -0.4357 | -0.3774 | 0.6211 | 1 |

**Supplementary Table 3: DN-4 and PainDETECT agreement**

| **Baseline** | PD = unlikely NP | PD = ambiguous NP | PD = likely NP | Total |
| --- | --- | --- | --- | --- |
| DN-4 = no NP | 47 (13%) | 23 (6%) | 22 (6%) | 92 (26%) |
| DN-4 = NP | 28 (8%) | 72 (20%) | 167 (47%) | 267 (74%) |
| Total* | 75 (21%) | 95 (26%) | 189 (53%) | 359 |

*4 patients had PD scores complete but were missing DN-4 (not in this table)

NP=neuropathic pain, PD=PainDETECT
